# Convergent evolution of highly reduced fruiting bodies in Pezizomycotina suggests key adaptations to the bee habitat

**DOI:** 10.1186/s12862-015-0401-6

**Published:** 2015-07-21

**Authors:** Anja Amtoft Wynns

**Affiliations:** Center for Social Evolution and Department of Plant and Environmental Sciences, University of Copenhagen, Thorvaldsensvej 40, Frederiksberg C, 1871 Denmark

**Keywords:** Ascosphaerales, Eremascaceae, Honey bees, Solitary bees, Yeasts, *Skoua*

## Abstract

**Background:**

Among the understudied fungi found in nature are those living in close association with social and solitary bees. The bee-specialist genera *Bettsia*, *Ascosphaera* and *Eremascus* are remarkable not only for their specialized niche but also for their simple fruiting bodies or ascocarps, which are morphologically anomalous in Pezizomycotina. *Bettsia* and *Ascosphaera* are characterized by a unicellular cyst-like cleistothecium known as a spore cyst, while *Eremascus* is characterized by completely naked asci, or asci not formed within a protective ascocarp. Before molecular phylogenetics the placement of these genera within Pezizomycotina remained tentative; morphological characters were misleading because they do not produce multicellular ascocarps, a defining character of Pezizomycotina. Because of their unique fruiting bodies, the close relationship of these bee-specialist fungi and their monophyly appeared certain. However, recent molecular studies have shown that *Bettsia* is not closely related to *Ascosphaera*.

In this study, I isolated the very rare fungus *Eremascus fertilis* (Ascomycota, Pezizomycotina) from the bee bread of honey bees. These isolates represent the second report of *E. fertilis* both in nature and in the honey bee hive. To establish the systematic position of *E. fertilis* and *Bettsia alvei*, I performed phylogenetic analyses of nuclear ribosomal LSU + SSU DNA sequences from these species and 63 additional ascomycetes.

**Results:**

The phylogenetic analyses revealed that *Eremascus* is not monophyletic. *Eremascus albus* is closely related to *Ascosphaera* in Eurotiomycetes while *E. fertilis* belongs in Myxotrichaceae, a putative member of Leotiomycetes. *Bettsia* is not closely related to *Ascosphaera* and like *E. fertilis* apparently belongs in Leotiomycetes. These results indicate that both the naked ascus and spore cyst evolved twice in the Pezizomycotina and in distantly related lineages. The new genus *Skoua* is described to accommodate *E. fertilis*.

**Conclusions:**

The naked ascus and spore cyst are both shown to have evolved convergently within the bee habitat. The convergent evolution of these unusual ascocarps is hypothesized to be adaptive for bee-mediated dispersal. Elucidating the dispersal strategies of these fungal symbionts contributes to our understanding of their interaction with bees and provides insight into the factors which potentially drive the evolution of reduced ascocarps in Pezizomycotina.

**Electronic supplementary material:**

The online version of this article (doi:10.1186/s12862-015-0401-6) contains supplementary material, which is available to authorized users.

## Background

The Ascomycota are an ecologically diverse group of fungi characterized by the production of meiospores in sac-like structures called asci. In the subphylum Pezizomycotina, asci are formed within a protective ascocarp, while in the basal lineages (Saccharomycotina and nearly all members of Taphrinomycotina) asci lack a protective covering and are called naked asci. Four major multicellular ascocarp types are recognized in the Ascomycota: apothecia (cup-shaped ascocarps with an exposed hymenium), perithecia (flask-shaped ascocarps with a pore through which ascospores are released), pseudothecia (ascocarps with asci contained in numerous locules), and cleistothecia (entirely closed ascocarps with no predefined opening and no regular arrangement of asci) [1, 2]. The traditional classification system of Pezizomycotina (Ascomycota) placed great emphasis on these ascocarp types. Subsequently, DNA sequence-based phylogenies have shown that similar ascocarp types have evolved multiple times in distantly related lineages [3, 4]. As more sequence data become available, it is increasingly clear that morphological convergence of ascocarp types is not uncommon, and that the evolution of ascocarps and modes of ascus and ascocarp dehiscence are frequently associated with common spore dispersal strategies [5, 6, 4].

Phylogenetic studies have identified the major lineages shaping the backbone of the fungal tree of life [7, 8, 4] but the placement of many taxa at and below the class level remains uncertain. Increased DNA sampling of understudied groups, especially those whose systematic placement is based on morphology, may be critical for enabling ancestral-state reconstructions of characteristics such as ascus-dehiscence type, ascocarp ontogeny, and lifestyle and spore dispersal strategies.

**Fig. 1:**
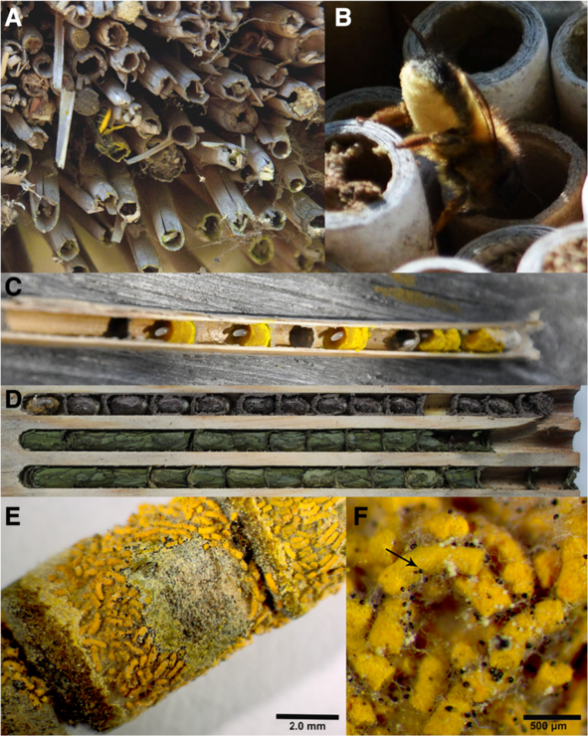
**a**. The solitary bee *Chelostoma florisomne* entering its nest — a *Phragmites* reed of a thatched roof. **b**. The solitary bee *Osmia bicornis* entering a commercial solitary bee nest made of cardboard tubes. **c**. A *Phragmites* reed peeled open to reveal the linear arrangement of brood cells built by *C. florisomne*, each containing pollen provisions and an egg. **d**. Opened man-made nest showing cocoons of *Osmia bicornis* (top row) and the leafcutting bee *Megachile centucularis* (bottom two rows). **e**. brood cell of *Osmia leaiana* containing a coccon and larval fecal pellets (orange cylinders) with *Ascosphaera* sporecysts. **f**. Close–up of larval fecal pellets from Fig. 1 **e** and intact spore cysts (arrow). Scale bars: E = 20 mm, F = 500 μm

**Fig. 2:**
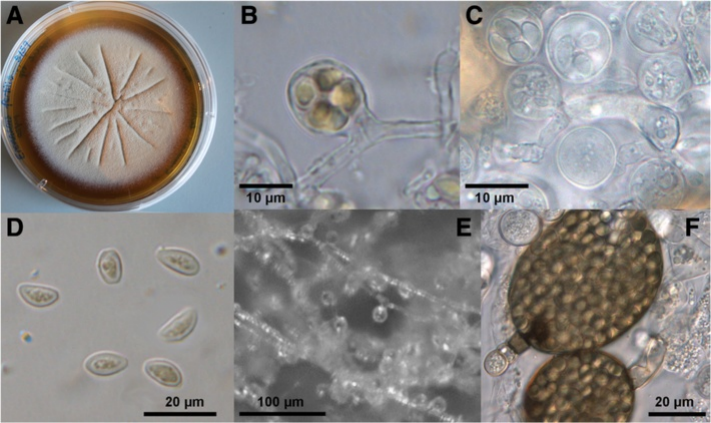
**a–e**: *Skoua fertilis*. A. Culture on MY20. **b–c**. Naked asci. **d**. Ascospores and pollen grains (orange). **e**. Hyphae bearing naked asci. **f**. Spore cysts of *Bettsia alvei* containing spherical ascospores. Scale bars: B–C = 10 μm, D = 20 μm, E = 100 μm, F = 20 μm

The three bee-specialist genera *Ascosphaera*, *Arrhenosphaera* (Ascosphaeraceae; Eurotiomycetes) and *Bettsia* (incertae cedis; formerly Ascosphaeraceae) are among the understudied and seldom-collected fungi. Within the bee habitat these fungi lead both saprotrophic and pathogenic lifestyles. They are found growing on pollen provisions, larval feces (Fig. 1e–f), cocoons, materials used by the bees to construct brood cells, and within bee larvae [9–15]. At least half of the species of *Ascosphaera* are bee brood pathogens infecting the larval stage and causing the bee disease commonly known as chalkbrood. In addition to sharing a specialized ecological niche [16], *Ascosphaera*, *Arrhenosphaera* and *Bettsia* are remarkable for their ascocarps (Figs. 1–2) which are morphologically anomalous in Pezizomycotina. Before DNA sequence data were available, the placement of Ascosphaeraceae among the Fungi and later within the Ascomycota was much debated [17–20]. Their odd ascocarps, called spore cysts, led some to suggest that members of Ascosphaeraceae might not be fungi at all or were possibly more closely related to genera in Entomophthoromycota (an early diverging lineage of Fungi) than to Ascomycota [18]. Spore cysts are completely closed ascocarps, which macroscopically resemble the multicellular cleistothecia of other fungi. However, the ontogeny and structure of a spore cyst and a cleistothecium differ. In a cleistothecium the asci develop within a multicellular ascocarp with variable complexity of the peridium, or ascocarp wall. In a spore cyst, evanescent asci develop within a single, enlarged cell which forms a cyst-like ascocarp with a simple acellular membranous peridium [17, 20].

Recent phylogenetic studies [21, 22] suggest that spore cysts have evolved at least twice and that *Bettsia* is more closely related to the class Leotiomycetes than to Ascosphaeraceae (Eurotiomycetes). Earlier phylogenetic studies supported a monophyletic Ascosphaeraceae; however, these studies included just two representatives of the family – *Ascosphaera apis* (Claussen) L.S. Olive & Spiltoir and *A. atra* Skou & K. Hackett (as *A. apis* in Berbee et al. 1995) – and not *Bettsia* [23, 24]. Because of the striking similarity of the ascocarps in *Bettsia* and *Ascosphaera*, and their shared predilection for the bee habitat, the position of *B. alvei* (Betts) Skou in the Leotiomycetes and the polyphyly of Ascosphaeraceae were formerly unsuspected. The affinity of the monotypic genus *Arrhenosphaera* to Ascosphaeraceae is based on the shared character of a spore cyst. Unfortunately, no holotype was designated in the protologue of *Arrhenosphaera cranei* Stejskal [9] and no additional collections or reports of the fungus have been made since its description as a problematic pathogen of honey bees in Venezuela in 1974.

The placement of Ascosphaeraceae in Eurotiales (Eurotiomycetidae) is based on similar early sexual development in *Monascus* and *Aspergillus* [17, 25], and is supported by DNA sequence-based phylogenies [3, 24]. These phylogenies also revealed a close relationship between Ascosphaeraceae and Eremascaceae, a family of just one genus, *Eremascus*. Ascosphaeraceae and *Eremascus* both lack hyphal fruiting bodies and for this reason were loosely referred to as ‘yeasts’ [26]. The shared simple ascocarp morphology of these two taxa was recognized before molecular data became available but their possible relatedness was doubted because of differences in their sexual systems [25, 27].

The genus *Eremascus* includes two species, *E. fertilis* Stoppel and *E. albus* Eidam, and is characterized by naked asci and a predilection for high sugar substrates [28]. Naked asci, with no interspersed sterile hyphae, occur elsewhere only within the early diverging lineages of the Ascomycota: the yeast-like Taphrinomycotina and the true-yeasts, Saccharomycotina [29]. The morphological similarity of *Eremascus* to the yeast-like fungi led some authors to believe that *Eremascus* should be placed among the Saccharomycetales, noting that the genus differs from other members of this group only in lacking asexual reproduction by budding or otherwise [30, 19]. Despite its unique fruiting body type within the Pezizomycotina, *Eremascus* has received little attention in phylogenetic studies. The prevailing view has been that *Eremascus* is monophyletic [31, 28], and its closest relative is Ascosphaeraceae [24, 32, 26]. The supposed monophyly of the genus is based on the shared character of completely naked asci, while the two species (*E. fertilis* and *E. albus*) differ in ascospore morphology and sexual development [27, 33].

Both *Eremascus* species are xerophiles thriving in conditions where water activity is low and solute concentrations are high [28]. The natural habitat of *Eremascus* remains elusive; both species are very rarely collected and have previously been isolated only from various high-sugar foods such as prunes, preserved fruit, shortcake, plum jam, mincemeat, honeycomb and on pollen [28, 34, 35]. In the process of studying the diversity of spore cyst fungi in the nests of solitary bees and in the hives of honey bees in Denmark, *Eremascus fertilis* was serendipitously isolated from the beebread of honey bee hives. This is the second report of *E. fertilis* in nature: the first report was in 1912 when A. Betts also observed the fungus growing on pollen in honey bee hives [35].

*Eremascus fertilis* has not previously been included in phylogenetic studies at the class level. In this study, I sequenced the large subunit (LSU) and small subunit (SSU) nuclear ribosomal DNA regions for *E. fertilis* and also for *Bettsia alvei*. These sequences were added to a large matrix of sequences from other Ascomycota, which was then analyzed both by maximum parsimony and Bayesian inference. Based on the resulting phylogenies, a theory for the adaptive significance of the spore cyst and naked ascus within the bee habitat is proposed.

## Methods

### Morphological study

Spore cysts and asci were mounted in water on glass slides. Light photomicrographs were made on an Olympus AX70 Provis light microscope. Herbarium acronyms follow those of Index Herbariorum [36] .

### Collection

Ninety-six solitary bee nests were placed at eight localities on the island of Sjælland, Denmark. The nest holes ranged from 6 to 9 mm in diameter (to attract different species of cavity-nesting bees) and had a length of 19.5 cm. The nests were placed 1–2.5 meters above the ground on the sides of buildings or below eaves with the entrances positioned to face southeast. Nests were opened and inspected annually from 2008 to 2012. Fungi growing on the pollen provisions, larvae, cocoons and nesting material were removed from a subset of the nests and identified. Overwintered honey bee (*Apis mellifera*) frames containing bee bread were collected in 2010 from three managed hives in Sjælland, Denmark. Bee bread with fungal growth resembling a spore cyst fungus was removed and the fungi isolated.

### Isolation and cultivation

Isolates obtained for sequencing in this study were grown at 18 °C on a solid medium of malt agar with 20 % dextrose (MY20). Spore cysts of *Bettsia alvei* and the asci of *Eremascus fertilis* were plated directly onto MY20 and kept at 18 °C. Once sufficient growth was present to verify the identity of the fungus, a single hyphal tip was cut from each culture and transferred to a fresh plate. Specimens are deposited in the herbarium of The Natural History Museum of Denmark (C), and isolate cultures, given the designation “KVL ##–##” (Table 1) are the part of the permanent fungal isolate collection of the Insect Pathology laboratory in the Department of Plant and Environmental Sciences at the University of Copenhagen (formerly Den Kongelige Veterinær og Landbohøjskole, or KVL), where they are stored at −80 °C.

**Table 1:** Collection information for new isolates of *Bettsia alvei* and *Skoua fertilis*

Taxon	Collection #	Isolate #	Habitat & substrate	Locality
*B. alvei*	5158	KVL 14–120	honey bee comb; bee bread	Frederiksværk, DK
*B. alvei*	5065	KVL 14–119	brood cell of *Osmia bicornis*; pollen	Taastrup, DK
*S. fertilis*	5160	KVL 10–10	honey bee comb; bee bread	Hundested, DK
*S. fertilis*	5159	KVL 10–09	honey bee comb; bee bread	Frederiksværk, DK

### Molecular study

For two strains of *Eremascus fertilis* and two strains of *Bettsia alvei* (Table 1), genomic DNA was obtained by picking up ascocarps and mycelium from isolate cultures and grinding them inside a 1.5 ml Eppendorf tube. DNA was then isolated using the Qiagen DNeasy Plant Mini Kit (Hilden, Germany) using the standard protocol and eluted in two separate 50 μl fractions to avoid over-dilution.

The LSU and SSU regions were each amplified by PCR. Primers LR0R and LR7 [37] were used to amplify 1.4 kb of LSU, and primers NS1 and NS4 [38] were used to amplify 1.1 kb of SSU. PCR reactions were prepared in 50 μl volumes containing 29.8 μl of sterile deionized water, 5 μl of *Taq* polymerase reaction buffer (Sigma®), 1.0 μl 10 mM dNTP, 3.0 μl 25 mM MgCl_2_, 0.2 μl *Taq* DNA polymerase (Sigma®), 5.0 μl each 10 μM primer and 1 μl of genomic DNA template. PCR was performed on a Biometra® thermocycler (Whatman) under the following conditions: for LSU: step 1) 1 min at 95 °C, 2) 1 min at 94 °C, 3) 30 sec at 51 °C, 4) 1 min at 72 °C, 5) return to step 2 34 times, 6) final step of 10 min at 72 °C; and for SSU: step 1) 1 min at 95 °C, 2) 1 min at 94 °C, 3) 30 sec at 51 °C, 4) 1 min at 72 °C, 5) 1 min at 94 °C, 6) 30 sec at 53 °C, 7) 65 sec at 72 °C, 8) return to step 2 34 times, 9) final step of 10 min at 72 °C. Samples were kept at 4 °C until electrophoresis was performed on 1 % agarose TAE gels and visualized with EZvision One® (Amresco). PCR products were cleaned using the Qiaquick® PCR purification kit (Qiagen) and were sent to Eurofins MWG Operon AG (Ebersberg, Germany) for sequencing. In addition to the amplification primers, LSU was sequenced with primers LR3R and LR5 [37], and SSU with primers NS2 [38] and SR7R (http://sites.biology.duke.edu/fungi/mycolab/primers.htm). Sequences were assembled using BioEdit [39].

**Fig. 3:**
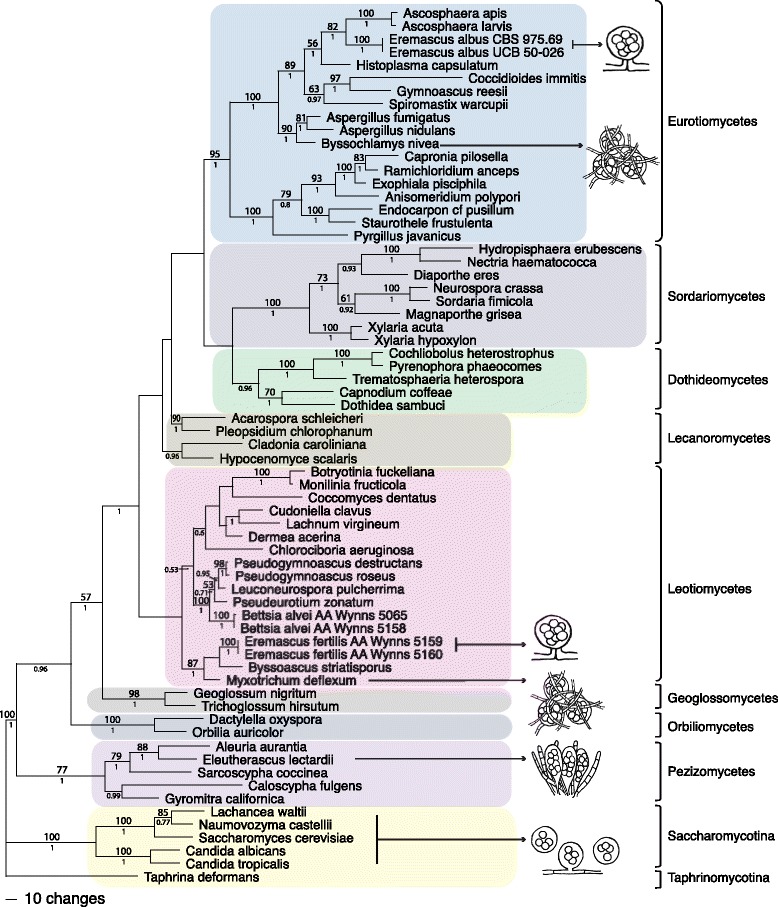
One of two equally most-parsimonious phylograms from a maximum parsimony analysis of nuclear ribosomal LSU and SSU DNA sequence data from 67 ascomycete fungi. Numbers above the branches are bootstrap support values (2000 replicates), and those below the branches are posterior probability values from a Bayesian analysis of the same data set. To the right of the arrows are diagrammatic illustrations of completely naked asci characteristic of *Eremascus albus*, *E. fertilis* (Pezizomycotina) and Saccharomycotina and nearly naked asci protected by hyphal wefts (*Gymnoascus*, *Myxotrichum*) or interspersed by sterile hyphae (*Eleutherascus*)

For each region, a data matrix that included sequences from two isolates of *Eremascus fertilis*, two isolates of *Bettsia alvei*, and 63 other ascomycetes (Table 2) was assembled and manually aligned in MEGA5 [40]. Taxon sampling was focused on genera with reduced fruiting bodies. Many of the sequences came from James et al. [8] and were downloaded from the AFToL website (aftol.org), while those from other studies [4, 41] came from GenBank. The LSU and SSU datasets were exported in NEXUS format and were combined in a single data file in PAUP* v. 4.0.10b [42]. The combined file was deposited in the Dryad Digital Repository, and can be accessed at http://dx.doi.org/http://dx.doi.org/10.5061/dryad.6s80j. This file was analyzed by maximum parsimony in PAUP*: using a random addition sequence and TBR swapping, 1000 heuristic replicates were performed, saving no more than ten best trees per replicate, followed by a final search of the saved trees. The file was also bootstrapped (2000 replicates) using the same search parameters except that only 10 heuristic replicates were performed per bootstrap replicate. A Bayesian analysis of the combined file was also performed using the program MrBayes v. 3.2 [43]. Based on the Akaike Information Criterion, the GTR + I + Γ model of DNA sequence evolution was selected as the best-fit model using the program Modeltest v. 3.06 [44]. A Markov chain Monte Carlo (MCMC) analysis was then run for 2,000,000 generations under the default settings, which was twice the number needed to keep the standard deviation of split frequencies below 0.01. Following Schoch et al. [4], Saccharomycotina was used as the outgroup for Pezizomycotina. Published single and multi-gene phylogenies [24, 45, 4] were followed in naming the major clades shown in Fig. 3.

**Table 2:** Isolates and/or voucher specimens and GenBank accession numbers for LSU and SSU sequences

Taxon	Isolate/strain/voucher specimen	GenBank ID/Sequence source	
		LSU rDNA	SSU rDNA
*Acarospora schleicheri*	VR 5-VII-98/30	AY640945	AY640986
*Aleuria aurantia*	OSC 100018	AY544654	NG_013139
*Anisomeridium polypori*	4237a	DQ782906	DQ782877
*Ascosphaera apis*	CBS 402.96	FJ358275.1	FJ358343
*Ascosphaera larvis*	ARSEF 7946	JX268535	JX268535
*Aspergillus fumigatus*	ATCC 1022/JCM1738	AY660917	AB008401
*Aspergillus nidulans*	ATCC 16855/ FGSC4	AF454167	U77377
*Bettsia alvei*	AA Wynns 5065 (C)	KR139932	KR139928
*Bettsia alvei*	AA Wynns 5158 (C)	KR139933	KR139929
*Botryotinia fuckeliana*	OSC 100012	AY544651	AY544695
*Byssoascus striatisporus*	CBS 642.66	AB040688	AJ315170
*Byssochlamys nivea*	CBS 100.11	FJ358279	FJ358345
*Caloscypha fulgens*	OSC 100062	DQ247799	DQ247807
*Candida albicans*	SC5314	AACQ01000290	AACQ01000290
*Candida tropicalis*	MUCL30002	AAFN01000124	M55527
*Capnodium coffeae*	CBS147.52	DQ247800	DQ247808
*Capronia pilosella*	W. Untereiner WUC28	DQ823099	DQ823106
*Chlorociboria aeruginosa*	OSC 100056	AY544669	AY544713
*Cladonia caroliniana*	F. M. Lutzoni 01-26-03.2	AY584640	AY584664
*Coccidioides immitis*	ATCC 28868	AAEC0200	AAEC0200
*Coccomyces dentatus*	OSC 100021	AY544657	AY544701
*Cochliobolus heterostrophus*	CBS 134.39	AY544645	AY544727
*Cudoniella clavus*	OSC 100054	DQ470944	DQ470992
*Dactylella oxyspora*	CBS 280.70	AY902790	AY902797
*Dermea acerina*	CBS 161.38	DQ247801	DQ247809
*Diaporthe eres*	CBS 109767	AF408350	DQ471015
*Dothidea sambuci*	DAOM 231303	AY544681	AY544722
*Eleutherascus lectardii*	CBS 626.71	DQ168334	DQ062997
*Endocarpon* cf. *pusillum*	S. Joneson 4028	DQ823097	DQ823104
*Eremascus albus*	UCB 50–026	M83258	GQ867787
*Eremascus albus*	CBS 975.69	FJ358283.1	FJ358348.1
*Eremascus* (*Skoua*) *fertilis*	AA Wynns 5159 (C)	KR139934	KR139931
*Eremascus* (*Skoua*) *fertilis*	AA Wynns 5160 (C)	HQ540515	KR139930
*Exophiala pisciphila*	W. Untereiner WUC 137	DQ823101	DQ823108
*Geoglossum nigritum*	OSC 100009	AY544650	AY544694
*Gymnoascus reesii*	CBS 259.61	FJ358284	FJ358349
*Gyromitra californica*	OSC 100068	AY544673	AY544717
*Histoplasma capsulatum*	AFTOL 1083	James et al. 2006 [8]	James et al. 2006 [8]
*Hydropisphaera erubescens*	ATCC 36093	AY545726	AY545722

## Results

### Collection

The predominant bee species in the solitary bee nests were *Osmia bicornis*, *O. leaiana* and *Megachile centuncularis* and *M. versicolor*. Details of the contents of individual cells were recorded for 1553 of the approximately 8000 brood cells observed. Of 1553 brood cells, 1429 contained cocoons, 80 contained uneaten pollen provisions, and 182 had spore cysts on the pollen provisions, brood cell building materials, larval feces, larvae, or on the cocoon surface. Eighteen brood cells contained chalkbrood caused by *Ascosphaera*. Thirty-five of the 80 brood cells containing uneaten pollen provisions had *Bettsia* and *Ascosphaera* growing on and between the pollen grains. *Bettsia* and *Eremascus* were found growing on the beebread of honey bee frames from two different localities (Table 1).

## Phylogenetic relationships of *Eremascus* and *Bettsia*

### Molecular study

The LSU matrix used for analysis included 862 characters, and the SSU matrix 1691 characters; the combined file thus included 2553 characters, of which 1762 were constant, 191 were variable but not parsimony-informative, and 600 were parsimony-informative. Two equally most-parsimonious trees of 3716 steps were recovered: a phylogram of one of these trees is shown in Fig. 3. Topologically, the parsimony and Bayesian trees [see Additional file 1] were similar to those of previous studies [8, 4] based on more regions. The analysis of the combined dataset placed *Eremascus fertilis* in a well-supported clade (bootstrap [BS] = 87; posterior probability [PP] = 1) with *Byssoascus striatosporus* (G.L. Barron & C. Booth) Arx and *Myxotrichum deflexum* Berk. *Eremascus albus*, the type species of the genus, was resolved in Eurotiomycetes, sister to *Ascosphaera* (BS = 82, PP = 1). *Bettsia* belonged to a fully supported clade including the leotiomycete genera *Pseudogymnoascus*, *Leuconeurospora* and *Pseudeurotium*. Although the Leotiomycetes clade did not receive statistical support, the Eurotiomycetes clade was well supported (BS = 95, PP = 1).

*Eremascus* is a polyphyletic taxon. A new genus is needed for *E. fertilis* which is evidently related to *Myxotrichum* and *Byssoascus*. The placement of *E. fertilis* with Myxotrichaceae is supported morphologically by its narrow ellipsoid ascospores and uncoiled suspensors resembling stipitate asci (Fig. 2b-c). Narrow ascospores and stipitate asci are characteristic of Myxotrichaceae but are anomalous in the Onygenales (Eurotiomycetes), where *E. fertilis* was formerly placed [46].

The placement of *Bettsia alvei* in Leotiomycetes (Fig. 3) agrees with recent studies [21, 22]. Thus, Ascosphaeraceae as traditionally understood is also polyphyletic and the family must be circumscribed more narrowly to include *Ascosphaera* and *Arrhenosphaera*, but not *Bettsia. Bettsia* is most closely related to *Pseudeurotium*, *Pseudogymnoascus* and *Leuconeurospora* in the family Pseudeurotiaceae. The cleistothecium of *Pseudeurotium* is formed by a cellular peridium [47] rather than a double membrane as in a spore cyst, and the ascomal ontogeny of *Pseudeurotium* [48] is markedly different from that of a spore cyst [49, 50]. However, *Pseudeurotium* and *Bettsia* are similar in that they both have prototunicate asci and globose ascospores.

#### Taxonomy

***Skoua*** A.A. Wynns, **gen. nov.**

Index Fungorum: IF551198

Type. *Skoua fertilis* (Stoppel) A.A. Wynns

##### Description

Ascomata absent. Asci borne laterally from undifferentiated hyphae, prototunicate, subglobose, stipitate-like from two suspensors. Ascospores ellipsoid, smooth, hyaline.

##### Etymology

The generic name *Skoua* commemorates the Danish bee pathologist J. P. Skou, in acknowledgement of his major contribution to our understanding of spore cyst fungi.

***Skoua fertilis*** (Stoppel) A.A. Wynns, **comb. nov.** Fig. 2a–e

Index Fungorum: IF551199

Basionym. *Eremascus fertilis* Stoppel, Flora 97: 332. 1907.

Neotype of *Eremascus fertilis* (here designated): DENMARK: Zealand, Frederiksværk. Isolated from overwintering bee bread of *Apis mellifera* hive 74 belonging to Christian Pedersen, collected by *A.A. Wynns 5159* (C). Index Fungorum: IF551197

##### Description

Naked asci subglobose, 9–12 μm, on average 11 μm. Ascospores 4–8 × 3–5 μm. Natural habitat on beebread inside the nests of *Apis mellifera*. In vitro growth at 18 °C, a low white mycelium on MY20, pale buff and radially sulcate with age (Fig. 2a), with abundant asci after four weeks. Conidia not observed.

Notes*—*In his description of *E. fertilis*, Stoppel [34] did not explicitly designate a holotype for this species; therefore, the ex-culture specimen *A.A. Wynns 5159* deposited in herbarium C is here designated as the neotype.

## Discussion

### Convergent evolution of reduced fruiting bodies

Peridia, or the protective structures enclosing asci and ascospores, are diverse among the cleistothecial fungi. Peridia range from completely closed structures composed of many cells (e.g., *Pseudeurotium*), to cottony or cage-like enclosures of hyphae (e.g., *Byssochlamys*, *Myxotrichum*, *Gymnoascus*), to simple hyphae interspersed among naked asci and not forming an enclosure (e.g., *Byssoascus*). The morphology of the peridium was once thought to indicate relatedness [51] but hyphal or mesh-like peridia, cephalothecoid peridia, and now membranous peridia are known to have independently evolved in unrelated lineages and in taxa associated with insects [4, 5].

The results of the phylogenetic analyses (Fig. 3) unambiguously show that *Eremascus fertilis* is excluded from the class Eurotiomycetes and is not closely related to the type species of the genus, *E. albus. Eremascus fertilis* is therefore transferred to the new genus *Skoua*. This genus is closest to Myxotrichacaeae, a family formerly placed in Eurotiomycetes but now considered to be a member of Leotiomycetes based on nrDNA-based phylogenies [45, 52]. Derived naked asci thus evolved at least twice within the Pezizomycotina: once in *Eremascus* (Eurotiomycetes) and once in *Skoua* (Leotiomycetes). Completely naked asci (i.e.,without any vestigial peridial hyphae) occur elsewhere only in the basal lineages of Ascomycota (Taphrinomycotina and Saccharomycotina) [53]. Unlike the yeasts and yeast-like filamentous fungi (e.g. *Symbiotaphrina*, *Aureobasidium*), asexual reproduction by budding, fission, or otherwise does not occur in *Eremascus* and *Skoua*; therefore, the only yeast-like character of *Skoua* and *Eremascus* is naked asci.

Several taxa in Pezizomycotina (e.g., *Byssochlamys*, *Gymnoascus*, *Pseudogymnoascus*, *Myxotrichum*, *Eleutherascus, Ascodesmis*) produce nearly naked asci that are enclosed by or interspersed with a loose network of hyphae (Fig. 3) known as a telaperidium or a reticuloperidium. Telaperidial and reticuloperidial ascomata are interpreted as loosely arranged cleistothecia or apothecia, a derived condition [46]. Interestingly, *Eremascus albus* and *Skoua fertilis* are allied to telaperidial and reticuloperidial taxa in both Eurotiomycetes (e.g., *Byssochlamys*, *Gymnoascus*) and Leotiomycetes (e.g., *Myxotrichum*, *Byssoascus*) (Fig. 3). More sequence data are needed to determine if the naked ascus in *S. fertilis* evolved by reduction from a reticuloperidium or if reticuloperidial taxa evolved from *Skoua*-like ancestors. Reduced fruiting bodies are also found in *Ascodesmis* and *Eleutherascus* in Pezizomycetes, the most basal clade of the Pezizomycotina. In *Ascodesmis* the fruiting body consists of a bundle of naked asci.

### Morphological convergence and dispersal

The discovery that the spore cyst and the naked ascus have each evolved twice in unrelated lineages of bee-associated fungi suggests that these reduced fruiting bodies are well adapted to the bee habitat. Convergent evolution of fruiting body types and ascus types in unrelated fungi with similar dispersal strategies has occurred repeatedly in the Pezizomycotina, e.g., ascomata with reticuloperidial or cephalothecoid peridia [5, 54–56]. Evanescent asci or asci which break down to release the ascospores passively evolved mutiple times in the Ascomycota and are correlated with insect dispersal [4, 57]. Similarly, the fragile peridium of the spore cyst fungi *Ascosphaera* and *Bettsia* and the evanescent naked asci of *Skoua* and *Eremascus* are interpreted as adaptive for dispersal by bees. Spore cysts break down when touched and dehisce with the activity of the bees, for example as bees move over and chew through the contents of brood cells in the process of emergence from their natal nests and during routine maintenance and construction of brood cells [58, 25, 59]. Disrupting the spore cyst membrane and subsequently picking up spores during emergence is a major contributor to the spread of chalkbrood in the managed solitary bee *Megachile rotundata* [60]. As the spore cysts are broken open, a sticky mucilage on the ascospores of *Bettsia* and *Ascosphaera* further ensures dispersal by the bee host [58, 25].

**Fig. 4:**
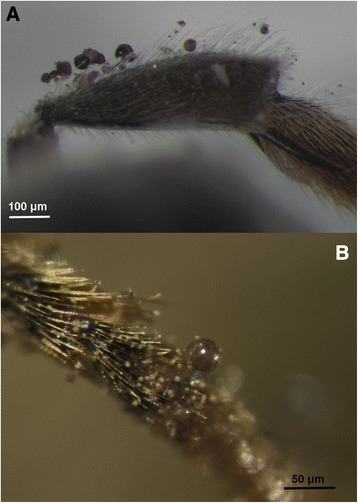
**a–b** Hind tibia of the solitary bee *Osmia bicornis* showing impaled spore cysts and sporeballs attached to hairs after contact with *Ascosphaera atra*. Scale bars: **a** = 100 μm, **b** = 50 μm

Spore cysts are not just broken open by the activity of bees, they also become impaled on their setae (Fig. 4). Impalement of fruiting bodies on arthropod setae is a spore dispersal mechanism found in other fungi, e.g. *Myxotrichum* (Leotiomycetes) and *Auxarthron* (Eurotiomycetes) [5]. In *Myxotrichum* and *Auxarthron* the mesh-like hyphal peridia function in attachment to the arthropod setae. Once impaled, ascospores fall through the holes in the mesh-like peridium and are dispersed as the insect (e.g., a fly) moves or flies around [5].

In this study, the peridia or membranous spore cyst walls of *Ascosphaera* and *Bettsia* growing within the nests of solitary bees remained intact in overwintering bee nests and in the absence of nesting activity. This is reminiscent of the spore dispersal strategy of Myxomycetes living in sheltered habitats. The peridium of Myxomycetes is dependent on the movement of invertebrates to disrupt the membrane, while in the absence of invertebrates the peridium remains intact for months [61].

The diversity and abundance of the spore cyst fungi is greater in the nests of solitary bees than in eusocial bees. The combination of the lack of social grooming and the nesting habits of solitary bees may contribute to the diversity and abundance of spore cyst fungi within the nests of these bees. In contrast to eusocial bees (e.g., honey bees), which may be active for months, solitary bees in temperate regions usually have only one generation per year and a relatively brief nesting and active period, sometimes as short as three weeks [62]. These nesting habits provide a stable environment for the slow-growing spore cyst fungi to establish and to develop mature ascocarps. On the other hand, because the active period of solitary bees is short compared with their eusocial relatives, the period for successful ascospore dispersal is much reduced. Ascospore release, coupled with the activity of the bees, maximizes spore dispersal of the fungus specifically by its host. Ensuring that ascospore release occurs in conjunction with the activity of the bees is particularly adaptive in the solitary bee habitat where the linear arrangement of brood cells means that siblings must pass through other brood cells to emerge from the nest (Fig. 1c–d).

Bee-mediated dispersal may be particularly critical for the spore cyst fungi and for *Eremascus* and *Skoua* because of their physiological requirements as xerophiles. *Eremascus* and *Bettsia* are included among the few xerophilic fungi called extreme xerophiles, meaning that they require rather than prefer a substrate with low water activity [63]. The bee habitat is a temporally static micro-environment that provides low water activity substrates, e.g., honey, bee bread, and pollen provisions, on which these slow growing xerophiles are able to thrive. A habitat with the combination of a temporally static environment and a provision of low water activity substrates is undoubtedly uncommon in nature and possibly unique to the nests of bees.

## Conclusions

Microbial symbionts in bee nests and within the bees themselves may play a major role in maintaining bee health and in disease defense [64–67]. However, characterization of the microbial community in even the best-studied bee system, the honey bee, remains understudied, and its beneficial potential poorly understood [65]. Even less is known about the fungi intimately associated with the many species of solitary bees that make up the majority of the 20,000 bee species [68]; the literature is sparse [69] and the subject almost entirely unexplored. Understanding the phylogenetic relationships of resident fungi within bee nests and their dispersal strategies may help to elucidate the role of these organisms in the bee habitat.

In this study, the systematic relationships of the bee specialist fungi are clarified and the monotypic genus *Skoua* is added to the microbial community associated with bees. The convergent evolution of spore cysts in *Bettsia* and *Ascosphaera*, and of naked asci in *Eremascus* and *Skoua*, is proposed to be adaptive for spore dispersal in the bee habitat. A taxonomic framework is provided for future studies of the understudied fungi occurring in this highly specialized niche.

### Availability of supporting data

The data set supporting the results of this article is available in the Dryad Digital repository; doi:http://dx.doi.org/10.5061/dryad.6s80j, http://dx.doi.org/http://dx.doi.org/10.5061/dryad.6s80j.

## Additional file

Additional file 1:
**A supplementary figure in PDF format (.pdf).** 50 %-majority rule consensus phylogram from a Bayesian analysis of nuclear ribosomal LSU and SSU DNA sequence data from 67 ascomycete fungi.
