# Supplementary material for: Convergent evolution of highly reduced fruiting bodies in Pezizomycotina suggests key adaptations to the bee habitat
Source: BMC Evol Biol. 2015 Jul 21;15:145. doi: 10.1186/s12862-015-0401-6 (PMC4509751; doi:10.1186/s12862-015-0401-6)
Supplement: Additional file 1: — A supplementary figure in PDF format (.pdf). 50 %-majority rule consensus phylogram from a Bayesian analysis of nuclear ribosomal LSU and SSU DNA sequence data from 67 ascomycete fungi. [file 12862_2015_401_MOESM1_ESM.pdf]

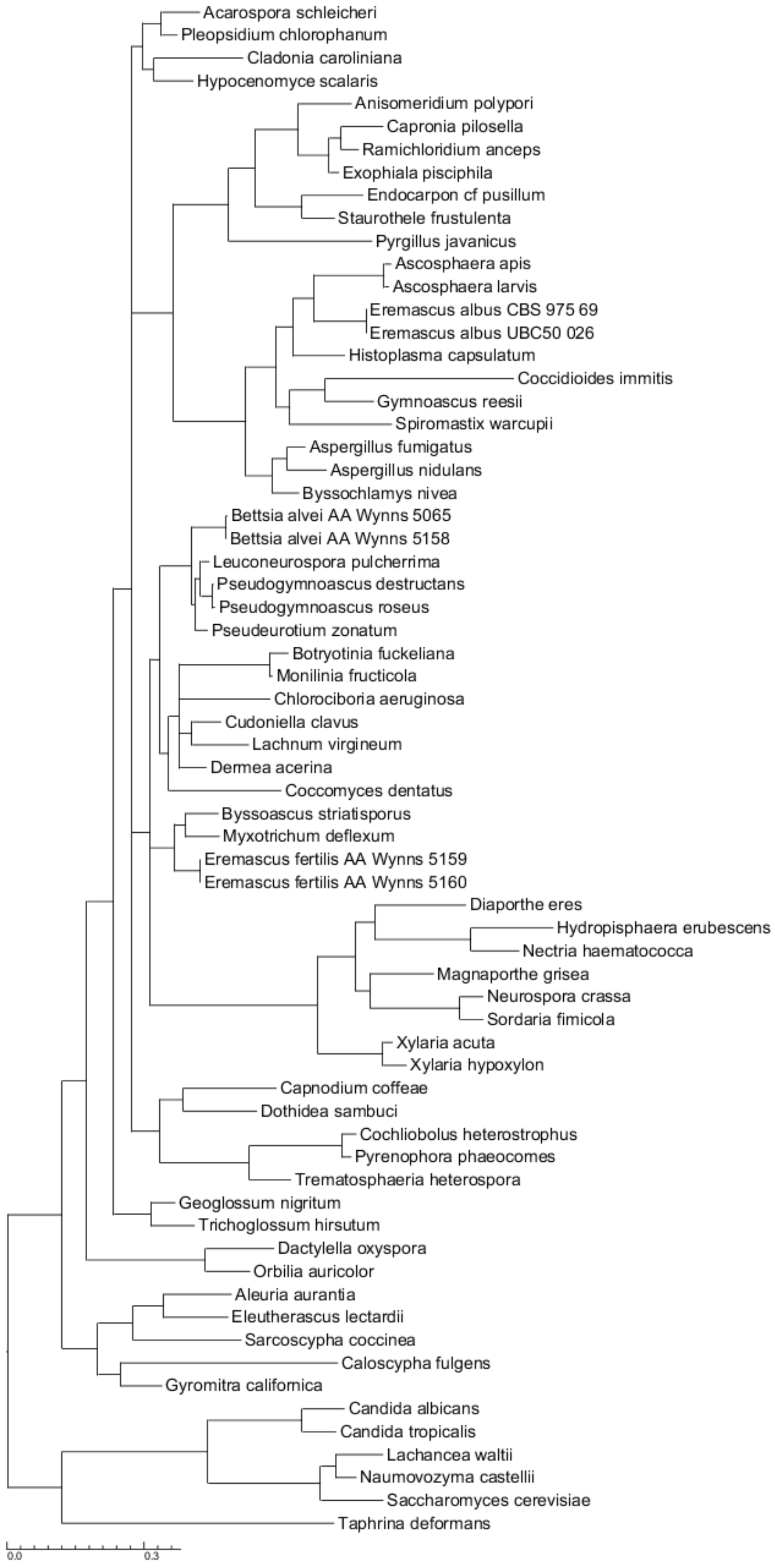

Additional file 1. 50%-majority rule consensus phylogram from a Bayesian analysis of nuclear ribosomal LSU and SSU DNA sequence data from 67 ascomycete fungi.
